# Express Detection of Pentachlorophenol as Dioxins Precursor in Natural Water

**DOI:** 10.1100/tsw.2002.209

**Published:** 2002-04-27

**Authors:** Vitalia S. Krikounova, Ramadan Abuknesha, Sergei A. Eremin

**Affiliations:** Faculty of Chemistry, M.V. Lomonosov Moscow State University, Russia; Life Sciences, King’s College London, University of London, UK

**Keywords:** pesticides, pentachlorophenol (PCP), dioxin, polarization fluoroimmunoassay, environmental monitoring

## Abstract

A rapid detection method for the pesticide pentachlorophenol (PCP) — polarization fluoroimmunoassay (PFIA) — in the dynamic range of 10–9,000 ppb was developed. PCP may form polychlorinated dibenzo-p-dioxins, making environmental monitoring of this compound an issue of great importance. In order to optimize the PFIA procedure, a number of fluorescein-labeled PCP derivatives and similar compounds (tracers) were synthesized, and the influence of their structure on PFIA characteristics was studied. Also, two antisera were tested in developing PFIA for PCP. The developed method is highly specific for PCP and can be used for its determination in water samples at a level down to 10 ppb. Total time of the assay for 10 samples is about 7 min. The assay provides a useful and a highly practical screening tool for the processing of large numbers of samples and for the preliminary estimation of potential dioxins contamination in water resources.

## INTRODUCTION

Pentachlorophenol (PCP) is a widely used substance as an insecticide in wood preservation and also in agriculture as a herbicide. It contaminates air at lumber mills and wood-treatment facilities, groundwater, surface water, fish habitats, and drinking water. PCP exposure can harm the liver, kidneys, blood, lungs, nervous system, immune system, and gastrointestinal tract. Moreover, PCP can form polychlorinated dibenzodioxins/furans, where the main product of its transformation is octachlorodibenzo-p-dioxin. Such processes take place under conditions such as in compost and sewage sludge[1,2]. In addition, dioxins can form in living cells from chlorophenols in peroxidase-catalyzed processes[3].

The most promising methods for PCP monitoring currently under development are antibody-based methods[4,5]. We have developed a polarization fluorescent immunoassay (PFIA), which is cost-effective, meets the requirements for water monitoring, and is highly specific, technically simple, and rapid (measurement of 10 samples takes 7–10 min). Water samples do not require any pretreatment. PFIA is a very convenient technique for environmental monitoring.

## EXPERIMENTAL METHODS/PROCEDURES

### Synthesis of Fluorescent Tracers

Fluorescein thiocarbamyl ethylene diamine (EDF) was synthesized as previously described from ethylene diamine and fluorescein isothiocyanate isomer I[6]. The EDF-PCP tracers were synthesized using the N-hydroxysuccinimide ester method. An amount of 8 mg (80 µmol) N-hydroxysuccinimide and 8 mg (40 µmol) of N,N′-dicyclohexylcarbodiimide were added to a solution of 20 mmol corresponding PCP-carboxy derivative in 0.2 ml of dimethylformamide. After 2 h stirring at room temperature, the solution was added to 5 mg (10 mmol) of EDF. Then the reaction mixture was stirred at room temperature for 3 h. Small portions of reaction mixture (50 µl) were separated by TLC using chloroform/methanol (4:1 v/v) as the eluent. The main yellow band at Rf 0.9 was isolated and stored in methanol at 4°C. The tracer solution was further diluted in 2.5 mM borate buffer and used for PFIA measurements.

### PFIA Procedure

Dilution curves were constructed as follows. Antiserum IgG fraction was diluted 1/50, 1/100, 1/200, up to 1/51,200 and incubated with the tracer solution in a total volume of 1 ml for 1 min at room temperature, followed by measurement of fluorescence polarization in 10 TDx glass cuvettes loaded into a “Photo Check” carousel. Assay-competitive calibration curves were constructed using an analyte stock solution of 1 g/l in methanol diluted with borate buffer to give 0.1, 1, 10, 100, 1,000, 10,000, and 100,000 µg/l. The standards (100 µl) were vortex-mixed with fluorescein-labeled hapten solution (0.5 ml) and an appropriate dilution of antiserum (0.5 ml). After 1 min incubation, fluorescence polarization values were measured in a polarization fluorimeter (TDx Analyzer, Abbot Laboratories). The same procedure was used for analysis of samples. The calibration curves were analyzed using a four-parameter equation using Origin 6.0 for Windows.

## RESULTS AND DISCUSSION

For the assay development, the antisera were obtained by immunization of rabbits with PCP-BSA and thio-PCP-BSA conjugates (Fig. 1). Antibodies obtained from PCP-BSA bind to the tracers better than other derivatives; titers of anti-PCP-BSA were about 1:20,000 while anti-thio-PCP-BSA had titers around 1:200. The former antiserum had also provided more sensitive calibration curves ($IC_{50}$ values for anti-PCP-BSA were about 1 ppm, for anti-thio-PCP-BSA about 10 ppm) and so was used further for assay development.

Tracers PCP-O-$(CH_2)_n$-EDF with different length of link between the target molecule (hapten) and fluorescent residue were synthesized (Fig. 2), and in the same way a tracer series from the aniline derivatives (PCP-N-$(CH_2)_n$-EDF) was synthesized. Also, heterologous tracers 2,4,5-T-EDF and 2,4,5-TP-EDF were investigated in the assay. Assay $IC_{50}$ values and limits of detection for different tracers are shown in Table 1. Tracer 2,4,5-T-EDF did not display any binding to the antise-

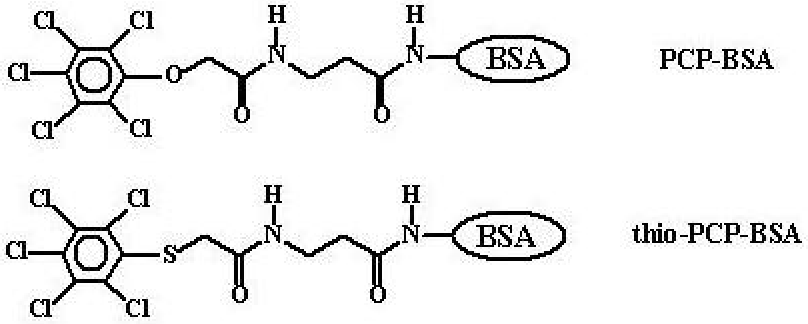

**FIGURE 1**. Structure of immunogens; the carrier protein is bovine serum albumin (BSA).

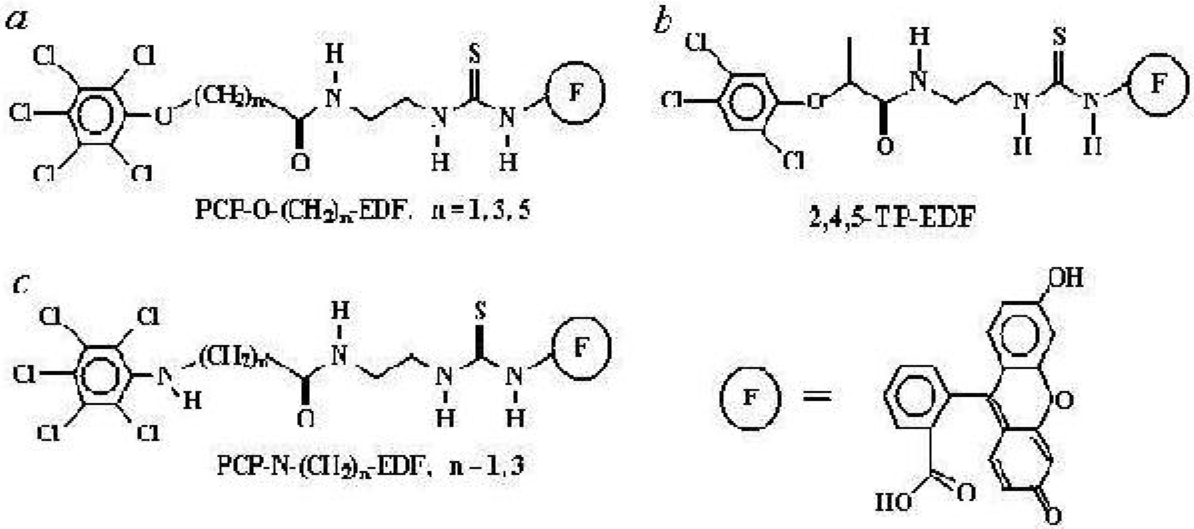

**FIGURE 2**. Structure of tracers: (A) homologous tracers with different linker length; (B) amino- substituted tracers with different linker length; (C) heterologous tracer 2,4,5-TP-EDF. F – fluorescein molecule.

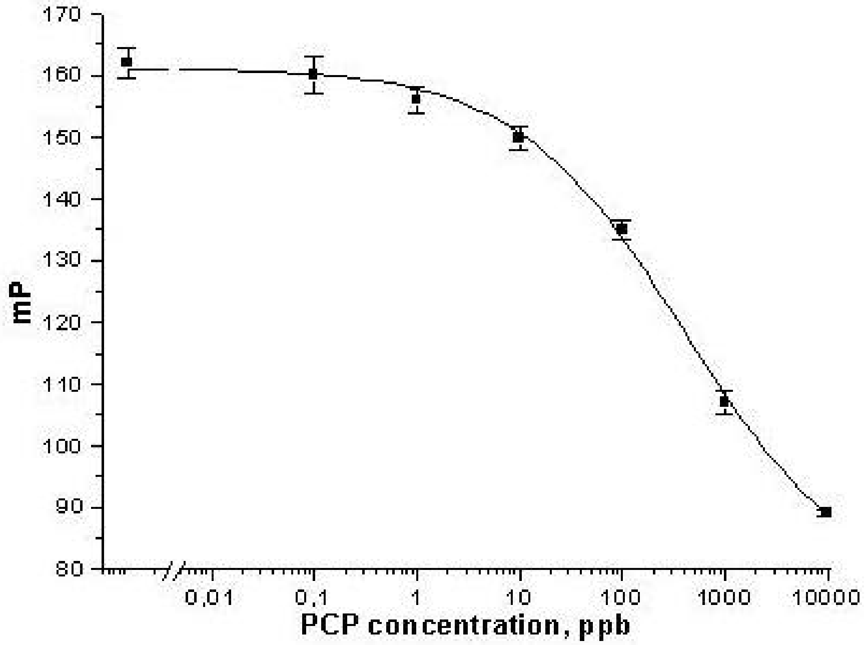

**FIGURE 3**. PFIA calibration curve for PCP (serum anti-PCP-BSA, tracer PCP-O-$CH_2$-EDF); the limit of detection is 10 ppb.

**TABLE 1**
**Titers of Antisera and PFIA Characteristics: $IC_{50}$ Values and Limits of Detection (DL) for Different Tracers and Serum Anti-PCP-BSA**

| Tracer | Serum dilution | $IC_{50}$, ppm | DL, ppb |
|---|---|---|---|
| PCP-O-$CH_2$-EDF | 1:20,000 | 0.5 | 10 |
| PCP-O-$(CH_2)_3$-EDF | 1:20,000 | 9.4 | 50 |
| PCP-O-$(CH_2)_4$-EDF | 1:20,000 | 9.3 | 90 |
| PCP-N-$CH_2$-EDF | 1:12,000 | 1.3 | 55 |
| PCP-N-$(CH_2)_3$-EDF | 1:12,000 | 1.1 | 100 |
| 2,4,5-TP-EDF | 1:300 | 1.8 | 85 |

rum. The best tracer for PFIA of PCP was the homologous tracer PCP-O-$CH_2$-EDF, which has the shortest spacer arm between hapten (PCP residue) and fluorescein. With the increase of the spacer arm length, the assay sensitivity decreased in both the phenol and anilin derivatives of PCP. 2,4,5-TP-EDF did not exhibit sufficiently high binding (serum titer was 1:300) but demonstrated an acceptable calibration curve, though less sensitive than homologous tracers. The assay limit of detection in the case of the most optimal tracer was 10 ppb; detectable concentration range or assay dynamic range was 10–9000 ppb.

The selectivity of the developed assays was also estimated. Cross-reactivity (%CR) was studied for related compounds (Table 2). Pentachlorophenoxyacetic acid displayed extremely high cross-reactivity percentage (2,076%). This was expected in view of its greater similarity to the immunogen than to the PCP molecule or due to the higher degree of homology. 2,4,5-trichlorophenoxyacetic (2,4,5-T) and 2,4,5-trichlorophenoxypropionic (2,4,5-TP) acids had %CR 8.3 and 4.7, respectively. The other compounds tested revealed much lower cross-reactivity.

The applicability of the developed methods was studied using field water samples. Recovery tests were carried out in river water, mineral water, and snow. Recoveries of all pesticides were in the range of 89–120% (Table 3).

To compare the method with the other immunochemical methods, competitive ELISA for PCP in water was developed by Van Emon and Gerlach[7]. It had a limit of detection of 30–40 ppb. In a

**TABLE 2**
**Cross-Reactivity Percentages (%CR) for Related Compounds**

| Cross-reagent | %CR |
|---|---|
| pentachlorophenol | 100 |
| pentaclorophenoxyacetic acid | 2076 |
| 2,4,5-trichlorophenol | 1.7 |
| 2,3,6-trichlorophenol | 1.1 |
| 2,4,5-trichlorophenoxyacetic acid | 8.3 |
| 2,4,5-trichlorophenoxypropionic acid | 4.7 |
| 3,5-dichlorophenol | 2.4 |
| 2,4-dichlorophenoxyacetic acid | 0.3 |
| 2-(2,4-dichloropro)-phenoxypropionic acid | 0.6 |
| 2,4-dichloro,5-fluorophenoxyacetic acid | 0.8 |
| 2-chloro,4-fluorophenoxyacetic acid | <0.1 |
| 2-(4-chloro,4-methyl)-phenoxyacetic acid | 0.7 |
| 2-(4-chloro,4-methyl)-phenoxypropionic acid | 0.7 |

**TABLE 3**
**Recovery Test in Natural Water. PCP Was Spiked into River Water, Mineral Water, and Snow Samples (n = 3, *p* = 0.95)**

| Added, ng/ml | Found | | | | | |
|---|---|---|---|---|---|---|
| | River water | | Mineral water | | Snow | |
| | ppb | % | ppb | % | ppb | % |
| 10 | 11±10 | 110±98 | 12±7 | 119±71 | 12±8 | 120±76 |
| 20 | 19±8 | 95±41 | 23±5 | 116±31 | 13±5 | 89±27 |
| 50 | 47±5 | 94±9 | 55±5 | 109±11 | 53±4 | 107±9 |
| 200 | 191±12 | 96±6 | 180±24 | 90±12 | 223±20 | 112±10 |
| 500 | 485±24 | 97±5 | 477±38 | 95±8 | 549±37 | 110±7 |
| 2000 | 1906±72 | 95±4 | 2211±107 | 111±5 | 2105±88 | 105±4 |

more recent source[8], use of ELISA was described for wastewaters, where PCP was detected at a level of 1–10 ppb. Thus the ELISA method has better sensitivity, but the advantage of the developed PFIA is rapidity (30 min for obtaining the calibration curve and 7 min for measurement of every 10 samples). This allows using it as a prescreening method for a great amount of environmental samples.

## CONCLUSION

The polarization fluorescent immunoassay for PCP is a suitable technique for routine testing of water contamination with the pesticide. The developed PFIA is less sensitive than the ELISA method[4,8] but is much more rapid and much simpler than all known bio and physical methods. The most sensitive assay was obtained by use of antisera obtained from immunogens with structures closest to PCP and with a homologous tracer with the shortest spacer. The developed PFIA is specific and can be applied for pesticide detection in natural water samples in the concentration range of 10–9,000 ppb. Analysis of water samples requires no pretreatment step.

The rapidity and simplicity of PFIA give it great perspective for wide application in environmental monitoring. As PFIA is a widely used technique for drugs and hormone tests, the same trend can be expected for pesticides and other environmental screening.

## ACKNOWLEDGEMENTS

This work have been supported by INCO-Copernicus grant ERBIC15-CT98-0910 (MEBFOOD) “Multichannel electrochemical biosensor for rapid food safety monitoring” and was presented at the CSIC/ESF workshop Analysis, Toxicity, and Biodegradation of Organic Pollutants in Groundwater from Contaminated Land, Landfills, and Sediments, Barcelona, Spain, November 8–10, 2001.

## References

1. Oberg, L.G., Anderson, R., and Rappe, C. (1993) De novo formation of PCDD/Fs in compost and sewage sludge — a status report. *Organohalogen Compd.* **11**, 297–302.
2. Schramm, K.W., Klimm, C., Henkelmann, K., and Kettrup, A. (1996) Formation of octa- and heptachlorodibenzo-p-dioxins during semi-anaerobic digestion of sewage sludge. *Organohalogen Compd.* **27**, 84–87.
3. Wittsiepe, J., Kullmann, Y., Schrey, P., Selenka, F., and Wilhelm, M. (1999) Peroxidase-catalysed in vitro formation of polychlorinated dibenzo-p-dioxins and dibenzofurans from chlorophenols. *Toxicol. Lett.* **106**, 191–200.
4. Wuske, T., Fittkau, I., Mahn, J., Polzius, R., and Manus, A. (1998) Pentachlorophenol detection at the source of emission. Sampling equipment and immunochemical analysis. *Anal. Chim. Acta* **359**, 321–328.

5. Li, M., Tsai, S.-F., Rosen, M.S., Wu, R.S., Reddy, K.B., DiCesare, J., and Salamone, S.J. (2001) Preparation of pentachlorophenol derivatives and development of a microparticle-based on-site immunoassay for the detection of PCP in soil samples. *J. Agric. Food Chem*. **49**, 1287–1292.
6. Pourfazaneh, M., White, G.W., Landon, J., and Smith, D.S. (1980) Cortisol directly determined in serum by fluoroimmunoassay with magnetizable solid phase. *Clin. Chem*. **26**, 730–733.
7. Van Emon, J.M. and Gerlach, R.W. (1992) Evaluation of a pentachlorophenol immunoassay for environmental water samples. *Bull. Environ. Contam. Toxicol.* **48**, 635–642.
8. Oubina, A., Puig, D., Gascón, J., and Barceló, D. (1997) Determination of pentachlorophenol in certified waste waters, soil samples and industrial effluents using ELISA and liquid solid extraction followed by liquid chromatograph. *Anal. Chim. Acta* ***346(1)****, 49–59.*
